# Unequal coverage of nutrition and health interventions for women and children in seven countries 

**DOI:** 10.2471/BLT.21.286650

**Published:** 2021-10-22

**Authors:** Phuong Hong Nguyen, Nishmeet Singh, Samuel Scott, Sumanta Neupane, Manita Jangid, Monika Walia, Zivai Murira, Zulfiqar A Bhutta, Harriet Torlesse, Ellen Piwoz, Rebecca Heidkamp, Purnima Menon

**Affiliations:** a Poverty, Health and Nutrition Division, International Food Policy Research Institute, 1201 I Street, NW, Washington DC, 20005, United States of America (USA).; b International Food Policy Research Institute, New Delhi, India.; c International Food Policy Research Institute, Kathmandu, Nepal.; d United Nations Children’s Fund, Regional Office for South Asia, Kathmandu, Nepal.; e Centre for Global Child Health, Hospital for Sick Children, Canada.; f Annapolis, USA.; g Department of International Health, Johns Hopkins Bloomberg School of Public Health, Baltimore, USA.

## Abstract

**Objective:**

To examine inequalities and opportunity gaps in co-coverage of health and nutrition interventions in seven countries.

**Methods:**

We used data from the most recent (2015–2018) demographic and health surveys of mothers with children younger than 5 years in Afghanistan (*n* = 19 632), Bangladesh (*n* = 5051), India (*n* = 184 641), Maldives (*n* = 2368), Nepal (*n =* 3998), Pakistan (*n* = 8285) and Sri Lanka (*n* = 7138). We estimated co-coverage for a set of eight health and eight nutrition interventions and assessed within-country inequalities in co-coverage by wealth and geography. We examined opportunity gaps by comparing coverage of nutrition interventions with coverage of their corresponding health delivery platforms.

**Findings:**

Only 15% of 231 113 mother–child pairs received all eight health interventions (weighted percentage). The percentage of mother–child pairs who received no nutrition interventions was highest in Pakistan (25%). Wealth gaps (richest versus poorest) for co-coverage of health interventions were largest for Pakistan (slope index of inequality: 62 percentage points) and Afghanistan (38 percentage points). Wealth gaps for co-coverage of nutrition interventions were highest in India (32 percentage points) and Bangladesh (20 percentage points). Coverage of nutrition interventions was lower than for associated health interventions, with opportunity gaps ranging from 4 to 54 percentage points.

**Conclusion:**

Co-coverage of health and nutrition interventions is far from optimal and disproportionately affects poor households in south Asia. Policy and programming efforts should pay attention to closing coverage, equity and opportunity gaps, and improving nutrition delivery through health-care and other delivery platforms.

## Résumé

**Objectif:**

Examen des inégalités et des écarts de perspectives dans la couverture commune des interventions en matière de santé et de nutrition dans sept pays.

**Méthodes:**

Nous avons utilisé les données des enquêtes démographiques et sanitaires les plus récentes (2015-2018) auprès de mères ayant des enfants de moins de 5 ans en Afghanistan (*n* = 19 632), au Bangladesh (*n* = 5051), en Inde (*n* = 184 641), aux Maldives (*n* = 2368), au Népal (*n =* 3998), au Pakistan (*n* = 8285) et au Sri Lanka (*n* = 7138). Nous avons estimé la couverture commune pour un ensemble de huit interventions dans le domaine de la santé et huit autres liées à la nutrition et nous avons évalué les inégalités au sein des pays en matière de couverture commune en fonction de la richesse et de la géographie. Nous avons examiné les écarts de perspectives en comparant la couverture des interventions en matière de nutrition avec celle des plateformes de prestation de soins correspondantes.

**Résultats:**

Seuls 15 % des 231 113 couples mère-enfant ont bénéficié des huit interventions sanitaires (pourcentage pondéré). Le taux de couples mère-enfant n’ayant bénéficié d’aucune intervention en matière de nutrition était le plus élevé au Pakistan (25 %). Les écarts de richesse (les plus riches par rapport aux plus pauvres) pour la couverture commune des interventions sanitaires étaient les plus importants au Pakistan (indice de pente des inégalités : 62 points de pourcentage) et en Afghanistan (38 points de pourcentage). Les écarts de richesse pour la couverture commune des interventions en nutrition étaient les plus élevés en Inde (32 points de pourcentage) et au Bangladesh (20 points de pourcentage). La couverture des interventions en matière de nutrition était plus faible que celle des interventions sanitaires associées, avec des écarts de perspectives s’étalant de 4 à 54 points de pourcentage.

**Conclusion:**

La couverture commune des interventions en matière de santé et de nutrition est loin d’être optimale et affecte de manière disproportionnée les ménages pauvres d’Asie du Sud. Les efforts en matière de politique et de programmation devraient viser à combler les écarts en matière de couverture, d’équité et de perspective et à améliorer la fourniture d’interventions de nutrition au travers de soins de santé et d’autres plateformes de prestation.

## Resumen

**Objetivo:**

Analizar las desigualdades y la falta de oportunidades en la cobertura conjunta de las intervenciones sanitarias y de nutrición en siete países.

**Métodos:**

Se utilizaron los datos de las encuestas demográficas y sanitarias más recientes (entre 2015 y 2018) de madres con hijos menores de 5 años en Afganistán (*n*= 19 632), Bangladesh (*n*= 5051), India (*n*= 184 641), Maldivas (*n*= 2368), Nepal (*n*= 3998), Pakistán (*n*= 8285) y Sri Lanka (*n*= 7138). Se calculó la cobertura conjunta de un grupo de ocho intervenciones sanitarias y ocho de nutrición y se evaluaron las desigualdades en la cobertura conjunta dentro del país según la riqueza y la geografía. Se analizaron las diferencias de oportunidad al comparar la cobertura de las intervenciones de nutrición con la cobertura de sus correspondientes plataformas de prestación de servicios sanitarios.

**Resultados:**

Solo el 15 % de 231 113 parejas madre e hijo recibieron las ocho intervenciones sanitarias (porcentaje ponderado). El porcentaje de parejas madre e hijo que no recibieron ninguna intervención de nutrición fue mayor en Pakistán (25 %). Las diferencias de riqueza (los más ricos frente a los más pobres) para la cobertura conjunta de las intervenciones sanitarias fueron mayores en Pakistán (índice de desigualdad de la pendiente: 62 puntos porcentuales) y en Afganistán (38 puntos porcentuales). Las diferencias de riqueza para la cobertura conjunta de las intervenciones de nutrición fueron mayores en India (32 puntos porcentuales) y en Bangladesh (20 puntos porcentuales). La cobertura de las intervenciones de nutrición fue menor que la de las intervenciones sanitarias asociadas, con diferencias de oportunidad que iban de 4 a 54 puntos porcentuales.

**Conclusión:**

La cobertura conjunta de las intervenciones sanitarias y de nutrición está lejos de ser óptima y afecta de manera desproporcionada a los hogares pobres de Asia Meridional. Los esfuerzos en materia de política y programación deben prestar atención a la reducción de las diferencias de cobertura, equidad y oportunidad, y a la mejora de la prestación de servicios de nutrición a través de la atención sanitaria y demás plataformas de prestación.

## ملخص

الغرض فحص التفاوتات وفجوات الفرص في التغطية المشتركة للتدخلات الصحية والغذائية في سبع دول .

الطريقة قمنا باستخدام بيانات من أحدث المسوحات السكانية والصحية (الفترة من 2015 إلى 2018) للأمهات ممن لديهم أطفال أصغر من 5 سنوات في أفغانستان (العدد = 19632)، وبنغلاديش (العدد = 5051)، والهند (العدد = 184641)، وملديف (العدد = 2368)، ونيبال (العدد = 3998)، وباكستان (العدد = 8285)، وسري لانكا (العدد = 7138). قمنا بتقدير التغطية المشتركة لمجموعة من ثمانية تدخلات صحية وثمانية تدخلات غذائية، وقمنا بتقييم تفاوتات التغطية المشتركة داخل كل دولة حسب الثروة والجغرافيا. وقمنا بفحص فجوات الفرص عن طريق مقارنة تغطية التدخلات الغذائية، في مقابل تغطية المنصات الخاصة بها لتقديم التدخلات الصحة.

النتائج 15% فقط من 231113 من ثنائيات الأمهات والأطفال تلقوا كل التدخلات الصحية الثمانية (النسبة المئوية المرجحة). أما النسبة المئوية لثنائيات الأمهات والأطفال الذين لم يتلقوا أي من التدخلات الغذائية، فقد كانت الأعلى في باكستان (25%). كانت فجوات الثروة (الأغنى مقابل الأفقر) للتغطية المشتركة للتدخلات الصحية هي الأكبر في باكستان (مؤشر الانحدار للتفاوت: 62 نقطة مئوية) وأفغانستان (38 نقطة مئوية). كانت فجوات الثروة في التغطية المشتركة للتدخلات الغذائية هي الأعلى في الهند (32 نقطة مئوية)، وبنغلاديش (20 نقطة مئوية). كانت تغطية التدخلات الغذائية أقل من تغطية التدخلات الصحية المناظرة لها، حيث تراوحت فجوات الفرص من 4 إلى 54 نقطة مئوية.

الاستنتاج التغطية المشتركة للتدخلات الصحية والغذائية بعيدة كل البعد عن المثالية، وتؤثر بشكل غير متناسب على الأسر الفقيرة في سبع دول. يجب أن تولي جهود السياسات والبرمجة اهتمامًا لسد فجوات التغطية، والإنصاف، والفرص، وتحسين تقديم خدمات التغذية من خلال منصات تقديم الرعاية الصحية وغيرها من الخدمات.

## 摘要

**目的:**

旨在研究七个国家在健康和营养干预全民覆盖方面的不均衡性和机会差距。

**方法:**

我们采用来自阿富汗 (*n *= 19,632)、巴基斯坦 (*n* = 8285) 、马尔代夫 (*n* = 2368)、孟加拉国 (*n* = 5051)、尼泊尔 (*n =* 3998)、斯里兰卡 (*n* = 7138) 和印度 (*n* = 184,641) 的有五岁以下儿童的母亲的最新（2015-2018 年）人口统计和健康调查数据。我们估计了一组八项健康干预和八项营养干预的全民覆盖范围，评估了国家内部的全民覆盖在财富水平和地理分布方面所体现的不均衡性。我们通过比较营养干预的覆盖范围和其对应的健康服务平台的覆盖范围来研究机会差距。

**结果:**

在 231,113 对母子中，只有 15% 的人接受了全部八项健康干预（权重比例）。巴基斯坦未接受营养干预的母子比例最高 (25%)。健康干预全民覆盖体现的财富差距（最富与最穷）最大的国家是巴基斯坦（不平等斜率指数：62 个百分点）和阿富汗（38 个百分点）。营养干预全民覆盖体现的财富差距最大的国家是印度（32 个百分点）和孟加拉国（20 个百分点）。营养干预覆盖范围低于相关的健康干预覆盖范围，机会差距从 4 到 54 个百分点不等。

**结论:**

健康和营养干预的全民覆盖率远未达到最佳值，且对南亚穷困家庭的影响尤为严重。政策和规划工作应注意缩小覆盖范围、平等和机会差距，并通过医疗保健和其他服务平台改善营养供给。

## Резюме

**Цель:**

Изучить неравенство и различия в возможностях совместного охвата мероприятиями в области здравоохранения и питания в семи странах.

**Методы:**

Авторы применили данные последних (2015–2018 гг.) демографических обследований и обследований состояния здоровья матерей с детьми младше 5 лет в Афганистане (*n* = 19 632), Бангладеше (*n* = 5051), Индии (*n* = 184 641), на Мальдивских островах (*n* = 2368), в Непале (*n* = 3998), Пакистане (*n* = 8285) и на Шри-Ланке (*n* = 7138). Авторы оценили совместный охват для набора из восьми мероприятий в области здравоохранения и восьми мероприятий в области питания, а также неравенство в совместном охвате внутри страны по благосостоянию и географическому признаку. Авторы изучили различия в возможностях, сравнив охват мероприятиями в области питания с охватом соответствующих платформ по предоставлению медицинской помощи.

**Результаты:**

Только 15% из 231 113 пар «мать-ребенок» участвовали во всех восьми мероприятиях в области здравоохранения (взвешенный процент). Процент пар «мать-ребенок», которые не участвовали ни в одном мероприятии в области питания, был самым высоким в Пакистане (25%). Разрыв в уровне благосостояния (самые богатые и самые бедные) для совместного охвата мероприятиями в области здравоохранения был самым большим в Пакистане (показатель крутизны неравенства: 62 процентных пункта) и Афганистане (38 процентных пунктов). Разрыв в уровне благосостояния для совместного охвата мероприятиями в области питания был самым высоким в Индии (32 процентных пункта) и Бангладеше (20 процентных пунктов). Охват мероприятиями в области питания был ниже, чем сопутствующими мероприятиями в области здравоохранения, с различиями в возможностях от 4 до 54 процентных пунктов.

**Вывод:**

Совместный охват мероприятиями в области здравоохранения и питания отнюдь не является оптимальным и непропорционально затрагивает неимущие домашние хозяйства в Южной Азии. При разработке политики и программ следует уделять внимание устранению пробелов в охвате, равенстве и возможностях, а также улучшению системы поставки продовольствия с помощью медицинских и других платформ.

## Introduction

Universal health coverage (UHC) is fundamental to achieving the sustainable development goals (SDGs), reducing inequalities and ensuring that no one is left behind.^1^ Timely delivery of a complete package of health and nutrition interventions to women during pregnancy and to children during early childhood is a goal of global and national health and nutrition initiatives. An index of co-coverage of health interventions has been proposed to track coverage and assess progress towards UHC.^2^^–^^4^ This widely used co-coverage index is a cumulative count of eight preventive health interventions that should be received by mothers and children.^4^^,^^5^ However, this index includes only one nutrition intervention and is thus an inadequate measure of nutrition intervention co-coverage.

Nutrition interventions are key components of UHC.^6^ There is evidence that a package of nutrition interventions, if scaled to 90% coverage, could reduce stunting by 20% and reduce infant and child mortality by 15%.^7^ However, in many countries on their way to achieving UHC, coverage of nutrition interventions is not keeping pace. To track the scale-up of nutrition interventions, a group of nutrition researchers proposed a set of 24 high-impact nutrition-specific interventions along with indicators for tracking their coverage.^8^ However, coverage data for nutrition interventions along the continuum of care remain scarce.

Many nutrition interventions can be delivered by existing health systems. For example, iron supplementation could be provided at antenatal care contacts during pregnancy; counselling and support for early initiation of breastfeeding could be provided by a skilled birth attendant at birth; and zinc could be delivered together with oral rehydration solutions for children having diarrhoea. Missed opportunities to deliver nutrition interventions through associated health platforms have been defined as opportunity gaps.^9^ Identifying opportunity gaps is important not just in terms of improving nutrition intervention coverage but because bridging such gaps may be achievable in the short or medium term with minimal additional inputs, as the delivery platform is already in place.

Afghanistan, Bangladesh, Bhutan, India, Maldives, Nepal, Pakistan and Sri Lanka are home to around 1.97 billion or 25% of the world’s population of 7.9 billion, almost 59 million of the world’s 151 million stunted children and 27 million of the world’s 51 million wasted children.^10^ Most countries have a robust health policy framework and national policies that commit to providing health and nutrition services for their populations.^11^ Nevertheless, health coverage and financial risk protection is low in these countries,^12^ and less is known about coverage of essential nutrition interventions. In this study, we examined co-coverage of pre-selected health interventions and nutrition interventions in seven countries. We also assessed geographical and wealth inequalities and quantified opportunity gaps between health platforms and their corresponding nutrition interventions.

## Methods

### Data sources

We used the most recent publicly available demographic and health survey data sets from seven countries (year): Afghanistan (2015), Bangladesh (2018), India (2016), Maldives (2017), Nepal (2016), Pakistan (2018) and Sri Lanka (2016). All demographic and health surveys included nationally and subnationally representative samples of ever-married women (aged 15–49 years) and children younger than 5 years; an exception was the survey in Bangladesh which included children younger than 3 years. Demographic and health survey questions are similar across countries, enabling cross-country comparisons and regional analyses. The number of households, women and children surveyed in each country are shown in Table 1. 

**Table 1:** Samples used in the latest demographic and health survey rounds for seven countries, 2015–2018

Country	Survey year	No. of households surveyed	Country population, in millions^a^	No. of ever-married women aged 15–49 years	No. of children aged < 5 years	No. of women aged 15–49 years with a live birth in the past 5 years
Afghanistan	2015	24 395	34.5	29 461	31 400	19 632
Bangladesh	2018	19 457	160.7	20 127	8 347	5 051^b^
India	2016	601 509	1 031	699 686	243 867	184 641
Maldives	2017	6 050	0.5	7 699	3 376	2 368
Nepal	2016	11 040	27.3	12 862	4 840	3 998
Pakistan	2018	14 540	212.3	15 068	12 803	8 285
Sri Lanka	2016	27 210	21.4	18 302	7 908	7 138

^a^ We collated population data using the World Bank’s total country population indicator for the year that is nearest to the demographic and health survey round.

^b^ For Bangladesh, the sample included women aged 15–49 years with a live birth in the last 3 years.

### Measures

We followed a multistep process to select the health and nutrition intervention coverage indicators. First, we conducted a comprehensive mapping of evidence-based health and nutrition interventions included in global recommendations and strategies,^7^^,^^8^^,^^13^^–^^19^ resulting in a list of 53 health and nutrition interventions (available in the data repository).^20^ We then selected a subset of eight health and eight nutrition interventions (Box 1) to include in our co-coverage calculations. The selection was based on four main criteria: (i) evidence of the benefit and the ability to scale up and deliver the intervention in low and middle-income countries; (ii) use of the intervention in previous UHC analyses;^2^^,^^4^^,^^21^ (iii) relevance of the intervention to outcomes across the continuum from pregnancy through early childhood (over a 1000-day period); and (iv) availability of data about the intervention in at least four of the seven countries analysed.

Box 1 **Selection of interventions for the study of co-coverage in Afghanistan, Bangladesh, India, Maldives, Nepal, Pakistan and Sri Lanka**

**Health interventions**

We selected eight health interventions based on recommendations from previous studies.^2^^,^^4^^,^^21^ The interventions included three interventions for mothers during pregnancy and delivery, three vaccines and vitamin A supplementation for the child, and safe water supply for the household.

Our list included: (i) four or more antenatal care visits; (ii) two tetanus toxoid injections for mothers; (iii) skilled birth attendant; (iv) child vitamin A supplementation; (v) child Bacillus Calmette–Guérin vaccination; (vi) child measles vaccination; (vii) child diphtheria-pertussis-tetanus vaccination; and (viii) household access to an improved source of drinking water.

**Nutrition interventions**

We found no previous studies which assessed co-coverage of nutrition interventions. We therefore selected a subset of interventions to reflect delivery across the continuum of care as suggested in the literature.^22^^,^^23^ We included two interventions during pregnancy, two interventions during birth, three interventions during childhood, and one intervention for the household. Data on these interventions are also the most commonly available across the countries based on our review of the literature (data repository).^20^

Our list included: (i) household consumption of iodized salt; (ii) consuming iron supplements for at least 100 days during pregnancy; (iii) receiving deworming tablets during pregnancy; (iv) early initiation of breastfeeding; (v) child weight measurement at birth; (vi) child iron supplementation; (vii) child vitamin A supplementation; and (viii) child deworming.

We retained child vitamin A supplementation in the health co-coverage calculations for comparability with previous studies, even though it is also a nutrition intervention. While we chose coverage indicators for which data were widely available, not all countries had information on all interventions (Table 2). For health interventions, Bangladesh lacked data on tetanus toxoid for mothers. Nutrition information was less complete. Iodized salt coverage data were lacking in Bangladesh, Maldives and Pakistan; data on deworming during pregnancy were not available in Bangladesh and Maldives; child iron supplementation data were lacking in Maldives; and coverage of iron supplementation during pregnancy was not available in Sri Lanka.

We calculated co-coverage as the number of health or nutrition interventions (ranging from zero to eight) that were received by individual mother–child pairs.^24^^,^^25^ For subnational variability and inequity analyses we also reported the proportion of mother–child pairs receiving four or more health and nutrition interventions (that is, at least half the set of interventions, as other studies have used).^4^ Additionally, we estimated the weighted median of health and nutrition co-coverage using population weights based on an individual country’s total female population aged between 15 and 64 years.^26^ For this analysis, we included only the youngest child aged between 12 and 59 months in the household because some interventions are only delivered late in infancy (vitamin A supplementation at 6 months, measles vaccine at 9 months or full immunization after 12 months).

### Statistical analyses

To examine subnational level geographical variability, we estimated weighted co-coverage means for health and nutrition interventions at the state or provincial level, accounting for sampling weights used in each survey. To assess inequalities in co-coverage for different wealth strata, we calculated a wealth index through principal component analysis using common household assets across country^27^^,^^28^ and used this to divide households into wealth quintiles. We then reported the percentage of mother–child pairs receiving at least four interventions in each wealth quintile using equiplots, which provide a visualization of inequalities within each country. We also examined absolute inequalities by the slope index of inequality (SII), which accounts for the entire distribution of the sample by wealth score.^24^ The index is the slope of the regression line, representing the absolute difference in the fitted value of the health indicator between the highest and the lowest quintile.

We also calculated the opportunity gap for three health–nutrition intervention pairs (Box 2). Examining these gaps can help to identify opportunities to influence health systems for nutrition more effectively. Data on oral rehydration solutions and zinc for treatment of diarrhoea were only available for a small subset of children with diarrhoea in the 2 weeks preceding the survey. We therefore used the data to examine the opportunity gaps, but did not include them in the co-coverage indicator to avoid sample loss. All analyses were performed using Stata version 16 (StataCorp, College Station, United States of America).

Box 2 **Selection of opportunity gap indicators for the study of co-coverage in Afghanistan, Bangladesh, India, Maldives, Nepal, Pakistan and Sri Lanka**

We selected three health–nutrition intervention pairs to assess opportunity gaps of interventions: (i) antenatal care visits and iron folic acid: % of mothers who received at least four antenatal care visits but did not consume ≥ 100 days of iron folic acid tablets during pregnancy; (ii) skilled birth attendance and early initiation of breastfeeding: % of mothers whose last delivery was attended by a skilled health professional but who did not practise early initiation of breastfeeding; (iii) oral rehydration solutions and zinc: % of children younger than 5 years with diarrhoea in the last 2 weeks who received oral rehydration solutions but did not receive zinc supplementation.

## Results

Our data included a total sample of 231 113 mother–child pairs: 19 632 in Afghanistan, 5051 in Bangladesh, 184 641 in India, 2368 in Maldives, 3998 in Nepal, 8285 in Pakistan and 7138 in Sri Lanka (Table 1).

### Prevalence of interventions

The weighted percentage coverage of health interventions during pregnancy, postpartum and in early childhood varied by intervention and by country (Table 2). Coverage of four or more antenatal care check-ups was highest in Sri Lanka (99%) and Maldives (82%). Coverage of a skilled birth attendant was also nearly universal in Sri Lanka and Maldives (each 99%), but only reached about half of pregnant women in Bangladesh and Afghanistan. Coverage of childhood interventions such as receiving vaccines was high in most countries (75–100%), except for Afghanistan (54–72%).

**Table 2:** Selected health and nutrition interventions used for co-coverage indicators, based on the latest demographic and health survey rounds for seven countries, 2015–2018

Intervention	Coverage of interventions, no. (%) of mother–child pairs						
	Afghanistan 2015 (*n* = 19 632)	Bangladesh 2018 *(n =* 5 051)	India 2016 (*n* = 184 641)	Maldives 2017 (*n* = 2 368)	Nepal 2016 (*n* = 3 998)	Pakistan 2018 (*n* = 8 285)	Sri Lanka 2016 (*n* = 7 138)
**Health interventions**							
Four or more antenatal care visits	3 534 (18)	2 374 (47)	94 167 (51)	1 942 (82)	2 799 (70)	4 308 (52)	7 067 (99)
Two tetanus toxoid injections for mothers	6 675 (34)	NA	155 098 (84)	1 137 (48)	2 639 (66)	5 220 (63)	3 355 (47)
Skilled birth attendant	10 601 (54)	2 677 (53)	153 252 (83)	2 344 (99)	2 679 (67)	6 048 (73)	7 067 (99)
Vitamin A supplementation for children	10 405 (53)	4 192 (83)	114 477 (62)	1 634 (69)	3 438 (86)	6 462 (78)	3 783 (53)
BCG vaccination	14 135 (72)	5 000 (99)	168 023 (91)	2 202 (93)	3 878 (97)	7 125 (86)	7 138 (100)
DPT vaccination	10 601 (54)	4 849 (96)	145 866 (79)	2 036 (86)	3 358 (84)	6 297 (76)	6 924 (97)
Measles vaccination	12 172 (62)	4 647 (92)	155 098 (84)	2 179 (92)	3 678 (92)	6 131 (74)	6 924 (97)
Access to improved drinking water	12 957 (66)	4 950 (98)	169 870 (92)	2 368 (100)	3 718 (93)	7 622 (92)	6 496 (91)
**Nutrition interventions**							
Household's consumption of iodized salt	11 190 (57)	NA	171 716 (93)	NA	3 758 (94)	NA	6 781 (95)
Iron supplements during pregnancy for at least 100 days	785 (4)	1 717 (34)	57 239 (31)	1 302 (55)	2 679 (67)	1 740 (21)	NA
Deworming during pregnancy	589 (3)	NA	33 235 (18)	NA	2 919 (73)	166 (2)	6 995 (98)
Birth weight measured	3 337 (17)	2 475 (49)	153 252 (83)	2 368 (100)	2 719 (68)	1 988 (24)	6 995 (98)
Early initiation of breastfeeding	8 245 (42)	3 081 (61)	81 242 (44)	1 634 (69)	2 239 (56)	1 574 (19)	6 424 (90)
Iron supplementation for children	1 374 (7)	354 (7)	49 853 (27)	NA	360 (9)	497 (6)	571 (8)
Vitamin A supplementation for children	10 405 (53)	4 192 (83)	114 477 (62)	1 634 (69)	3 438 (86)	6 462 (78)	3 783 (53)
Deworming for children	3 926 (20)	2 273 (45)	64 624 (35)	1 397 (59)	2 759 (69)	1 408 (17)	5 211 (73)

BCG: Bacillus Calmette–Guérin; DPT: diphtheria-pertussis-tetanus; NA: data not available.

Note: The values are weighted counts and percentages calculated using population weights based on an individual country’s total female population aged between 15 and 64 years. *n* is the total sample of mothers with children younger than 5 years.

Coverage of nutrition interventions was much lower than coverage of health interventions (Table 2). Consuming at least 100 days of iron tablets by pregnant women was low for most countries (4–34%), except Maldives (55%) and Nepal (67%). Similarly, child iron supplementation was very low in all countries (6–27%). Measuring child’s weight after birth varied substantially, from 17% in Afghanistan and 24% in Pakistan to 98% in Sri Lanka and 100% in Maldives.

### Intervention co-coverage

**Fig. 1:**
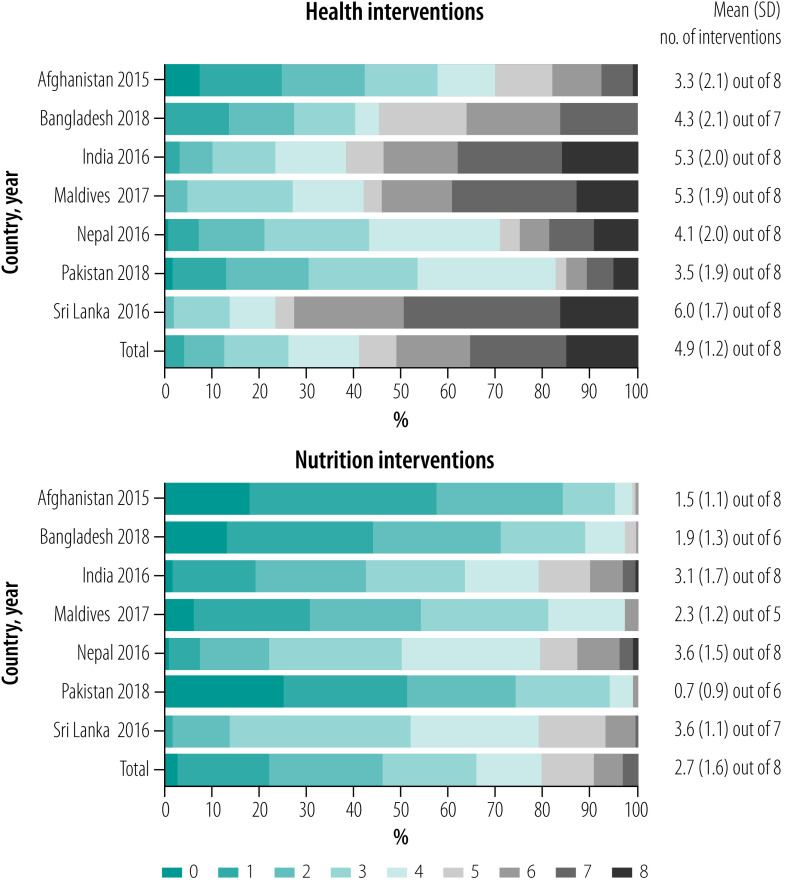
Number of health and nutrition interventions received by mothers and their youngest child by country and year, seven countries, 2015–2018

On average, mother–child pairs received three to five of the eight health interventions in most countries, except in Sri Lanka where they received six interventions (Fig. 1). The percentage of mother–child pairs who did not receive any health interventions varied from 0% in Sri Lanka and Maldives to 7% in Afghanistan. On average, 15% of the population across the countries received all eight health interventions, ranging from 0% in Bangladesh to 16% in India and Sri Lanka. Afghanistan had the highest percentage of children who received fewer than four interventions (57%), followed by Pakistan (53%) and Nepal (42%).

SD: standard deviation.

Notes: Health interventions included at least four antenatal care visits, at least two tetanus vaccinations, skilled birth attendant, child vitamin A supplementation, child Bacillus Calmette–Guérin vaccination, measles and diphtheria-pertussis-tetanus vaccination, and household with improved drinking water source. Data were unavailable for tetanus in Bangladesh. Nutrition interventions included household iodized salt, iron supplementation during pregnancy, deworming in women, early initiation of breastfeeding, child weight measured at birth, child iron supplementation, child vitamin A supplementation, and child deworming. Data were unavailable for some indicators in some country rounds: Bangladesh 2018: iodized salt and deworming in women; Maldives: iodized salt, deworming in women, child iron supplementation; Pakistan: iodized salt; Sri Lanka: iron supplementation during pregnancy.

Compared with health interventions, coverage of nutrition interventions was much lower. On average, a mother–child pair received between one and three nutrition interventions (Fig. 1). The proportion of mother–child pairs who did not receive any nutrition interventions was highest in Pakistan (25%) and Afghanistan (18%). Almost no mother–child pairs received all eight nutrition interventions.

### Subnational variability

We found wide variations in health intervention co-coverage (at least four interventions) across different regions of Afghanistan (ranging from 0.3% to 62% per region), India (42–96%) and Pakistan (15–59%). More details are in the data repository.^20^ There was less heterogeneity in co-coverage of health interventions in Bangladesh, Maldives and Sri Lanka.

Co-coverage of at least four nutrition interventions was below 10% for almost all regions in Afghanistan and Pakistan, and slightly higher in Bangladesh (6–14%) and Maldives (12–31%). The greatest subnational heterogeneity in co-coverage of nutrition interventions was found in India (ranging from 10–80% across states).

### Wealth inequity

**Fig. 2:**
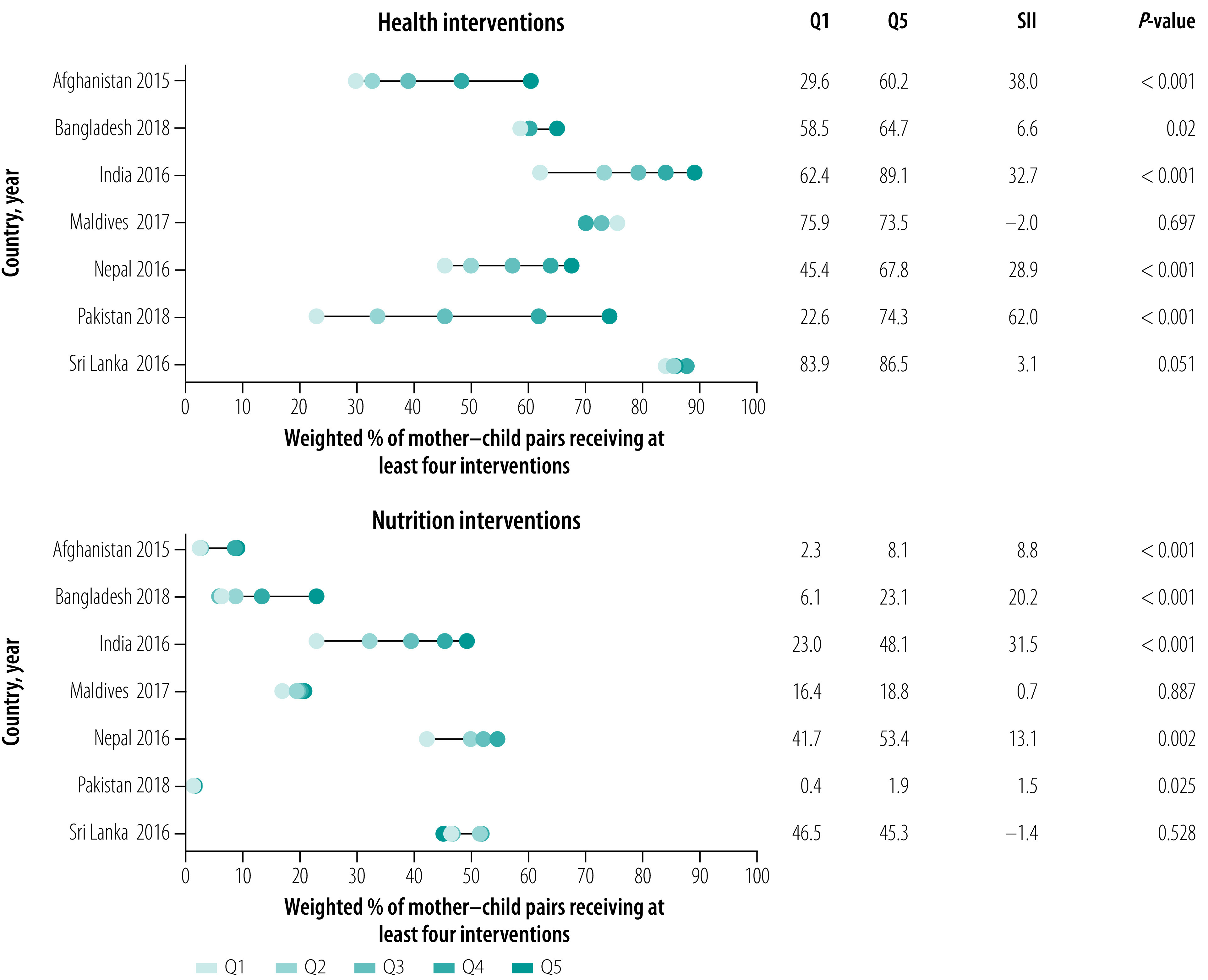
Wealth inequality in co-coverage of health and nutrition interventions, seven countries, 2015–2018

Co-coverage of health interventions was, as expected, lower among poorer than wealthier households across all countries except Maldives and Sri Lanka, as shown by the equity plots and positive SII (Fig. 2). The wealth gaps (richest versus poorest) were largest for Pakistan (SII: 62 percentage points), followed by Afghanistan (SII: 38 percentage points), India (SII: 33 percentage points) and Nepal (SII: 29 percentage points).

Q: quintile; SII: slope index of inequality.

Notes: Health interventions included at least four antenatal care visits, at least two tetanus vaccinations, skilled birth attendant, child vitamin A supplementation, child Bacillus Calmette–Guérin vaccination, measles and diphtheria-pertussis-tetanus vaccination, and household with improved drinking water source. Data were unavailable for tetanus in Bangladesh. Nutrition interventions included household iodized salt, iron supplementation during pregnancy, deworming in women, early initiation of breastfeeding, child weight measured at birth, child iron supplementation, child vitamin A supplementation, and child deworming. Data were unavailable for some indicators in some country rounds: Bangladesh 2018: iodized salt and deworming in women; Maldives: iodized salt, deworming in women, child iron supplementation; Pakistan: iodized salt; Sri Lanka: iron supplementation during pregnancy.

Co-coverage of nutrition interventions also exhibited pro-rich inequality patterns where coverage was higher among the rich than the poor (Fig. 2). The wealth gap was most prominent for India (SII: 32 percentage points), followed by Bangladesh (SII: 20 percentage points). Among the four countries with less variability between quintiles, Pakistan and Afghanistan had extremely low co-coverage (1–8%), Maldives had low co-coverage (16–19%) and Sri Lanka had medium co-coverage (45–47%).

### Opportunity gaps

**Fig. 3:**
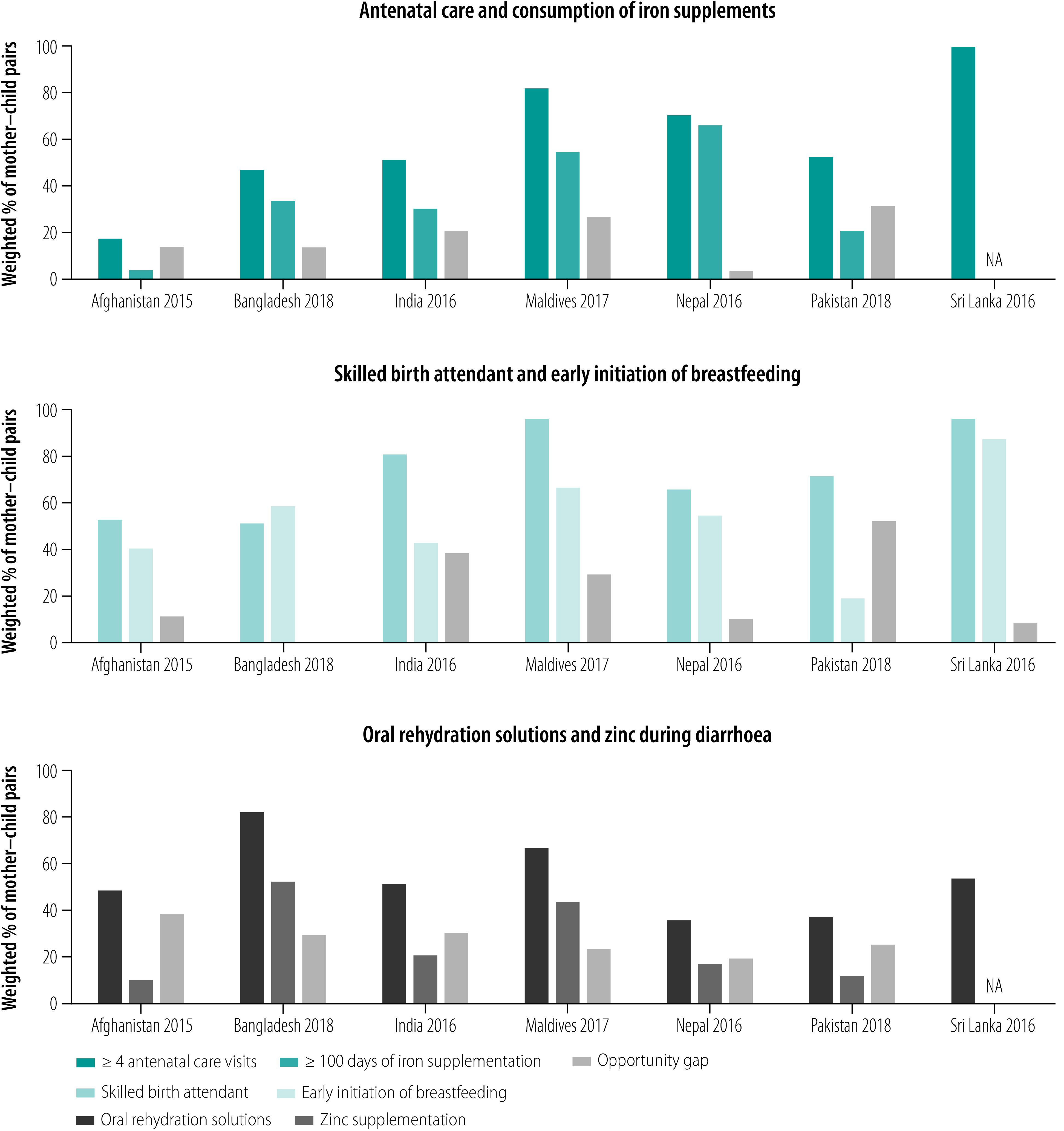
Opportunity gaps between coverage of health interventions and corresponding nutrition interventions, seven countries, 2015–2018

Coverage of nutrition interventions was lower than coverage of health-system delivery platforms for all countries (Fig. 3). The absolute opportunity gap between having four or more antenatal care visits and consuming 100 or more days of iron tablets ranged from 4 percentage points in Nepal to 31 percentage points in Pakistan (Fig. 3). The coverage of early initiation of breastfeeding was much lower than skilled birth attendant reach for Pakistan (73 versus 19 percentage points), India (83 versus 44 percentage points), and Maldives (99 versus 69 percentage points) (Fig. 3). Many children with diarrhoea received oral rehydration solutions but did not receive zinc, with opportunity gaps ranging between 19 percentage points and 38 percentage points (Fig. 3).

NA: data not available.

## Discussion

According to the most recent demographic and health survey data, south Asian countries are still far from achieving universal coverage of recommended health and nutrition interventions. On average, only 0–16% of mother–child pairs in countries in the region received all eight health interventions and close to zero mother–child pairs received all eight nutrition interventions. Co-coverage was lower among people from poor households and varied substantially at the subnational level. Coverage of nutrition interventions was consistently lower than their associated health interventions, highlighting gaps in opportunities to deliver different interventions at the same time. These gaps need to be addressed to achieve universal coverage of both health and nutrition interventions within the health-care delivery system.

Estimating co-coverage of health and nutrition interventions has important implications for health programmes as it helps us to understand whether essential interventions reach different segments of the population. Co-coverage measures also inform policy and programmatic interventions to address inequalities. Only a small proportion of mother–child pairs received all eight health interventions (16% in India and Sri Lanka and less than 10% in other countries). These findings are concerning as many mother–child pairs missed recommended interventions during pregnancy, birth or childhood life stages. Across the continuum of care, we found a wide variation in coverage and co-coverage levels within and across countries. Afghanistan had the lowest coverage for almost all interventions, which may be partly explained by the access challenges arising from conflicts and security issues,^29^ together with poor governance and frail accountability mechanisms.^11^ We observed large wealth inequalities in co-coverage among countries with lower co-coverage of health interventions (Afghanistan and Pakistan) but no wealth gaps for countries with higher co-coverage (Maldives and Sri Lanka). These findings are consistent with previous studies,^12^^,^^29^ and align with global observations that overall inequality tends to decrease as coverage increases.^4^ India and Nepal followed a different pattern, with high inequality but more than 50% coverage, which may reflect geographical variations. Findings from an analysis of UHC revealed several challenges including conflicts between political aspirations and government expenditure as well as insufficient progress for the neediest populations.^11^

The importance of nutrition interventions within health systems is often ignored.^9^ In our analyses, for the countries with available data for all eight health and eight nutrition interventions (Afghanistan, India and Nepal), a mother–child pair on average received between three and six health interventions, but only between one and three nutrition interventions. At the health-service delivery level, too few trained service providers, inadequate supplies and low quality of care have been identified as barriers to providing timely nutrition services to all women and children across the wealth spectrum.^11^^,^^25^^,^^30^ Nepal is the only country where the co-coverage was similar for health and nutrition interventions. Nepal has been more successful than other countries in increasing coverage of nutritional interventions such as iron folic acid supplementation. The success is mainly due to the use of community-based delivery systems and service providers with strong social mobilization to reach women, as well as decentralized governance structures.^31^

In many low-resource settings, several interventions are delivered together during appropriate contacts with women and children to improve delivery efficiency and increase coverage. Consistent with global evidence,^9^ we found large opportunity gaps in all countries where coverage of nutrition interventions was consistently lower than the reach of health delivery platforms. Thus, while closing wealth coverage gaps for health interventions is important, it is also necessary to increase co-coverage of nutrition interventions by using the full potential of health systems. A review of 45 studies on integrating nutrition into health systems documented various health programmes with the potential to include nutrition-specific interventions.^32^ Such programmes include integrated community case management and integrated management of childhood illness, child health days, immunization and early child development. Although there is evidence of well-integrated programmes relating to service delivery and the health workforce, many challenges remain. Issues related to governance, information systems, finance, supplies and technology call for better planned and better designed programmes to address the health-system faults responsible for the opportunity gaps.^6^^,^^32^ Beyond the traditional health platforms, other potential delivery channels for nutrition interventions vary by intervention type and could include agriculture, food systems and social protection programmes.^33^

Our analysis highlights the limited data available on nutrition intervention coverage. Demographic and health survey data on high-impact nutrition interventions are scarce, with data only available for eight of 24 recommended nutrition interventions that should be tracked.^8^ Scarcity of nutrition coverage data along the continuum of care prevents policy-makers from tracking progress and scaling up effective nutrition interventions. We echo previous calls for investment from donors and policy-makers in collecting data on nutrition intervention coverage and quality.^8^^–^^10^ On a positive note, consultation with technical experts, country users and survey representatives has identified priority data needs to strengthen nutrition content in the next (eighth) phase of demographic and health surveys. Updates have been made to include additional data on coverage of nutrition counselling, food and cash assistance, anthropometry assessment, and the sources and types of iron-containing supplements consumed, among others.^34^^,^^35^

The strengths of our analysis include the large and nationally representative samples which permitted estimates of coverage of individual interventions and co-coverage of multiple interventions at national and subnational levels. Data collection for all countries used standard methods with similar questionnaires, allowing for cross-country comparisons. The results provide evidence for a proposed nutrition co-coverage index, which could be used for tracking progress towards UHC and nutrition targets. We also identified several opportunity gaps that should be addressed to increase coverage and improve the efficient delivery of interventions. 

However, our study has some limitations. The primary limitation was lack of data on most of the recommended nutrition interventions; filling these data gaps will lead to a more informative nutrition co-coverage index in the future. Second, the demographic and health surveys obtain information on coverage by self-reported data on only the youngest child with a recall period of 3–5 years, which misses data on other children and could be subject to recall bias.^36^^,^^37^ Our analyses did not address effective coverage, that is, the adequacy and quality of care provided during contacts with health workers,^38^ or the effective coverage index and its associated potential health gains.^39^ Measurement of quality of care continues to be a challenge in large-scale surveys. Finally, our study focused mainly on nutrition-specific interventions delivered through the health sector. To accelerate progress, continued focus is needed on actions across different sectors (including education, empowerment, food systems, social protection and hygiene).^40^^,^^41^

In conclusion, with fewer than 10 years remaining to achieve the SDGs, significant progress is still required to achieve UHC. While some countries such as India, Maldives and Sri Lanka, are reaching their populations with essential health services, the region is diverse, and large inequities persist within all countries. The co-coverage of nutrition interventions is low and lags behind health co-coverage everywhere. Given the importance of nutrition for achieving multiple SDGs, we call for immediate attention to close opportunity gaps and expand nutrition delivery through health and other delivery platforms. We also call for robust health and nutrition coverage data to enable progress-tracking, given the lack of recent information for this analysis. Future work should examine lessons for closing inequities in health and nutrition intervention delivery in the region along the continuum of care.
